# Bit by Bit: The Darwinian Basis of Life

**DOI:** 10.1371/journal.pbio.1001323

**Published:** 2012-05-08

**Authors:** Gerald F. Joyce

**Affiliations:** 1 Department of Molecular Biology, Skaggs Institute for Chemical Biology, The Scripps Research Institute, La Jolla, California, United States of America; 2 Department of Chemistry, The Scripps Research Institute, La Jolla, California, United States of America

## Abstract

All known examples of life belong to the same biology, but there is increasing enthusiasm among astronomers, astrobiologists, and synthetic biologists that other forms of life may soon be discovered or synthesized. This enthusiasm should be tempered by the fact that the probability for life to originate is not known. As a guiding principle in parsing potential examples of alternative life, one should ask: How many heritable “bits” of information are involved, and where did they come from? A genetic system that contains more bits than the number that were required to initiate its operation might reasonably be considered a new form of life.

## Abstract 2

Why do we have so much trouble distinguishing life from non-life, and distinguishing our biology from another? When in doubt, one should count the “bits” of heritable information.

Thanks to a combination of ground- and space-based astronomical observations, the number of confirmed extrasolar planets will soon exceed 1,000. An increasing number of these will be said to lie within the “habitable zone” and even be pronounced as “Earth-like.” Within a decade there will be observational data regarding the atmospheric composition of some of those planets, and just maybe those data will indicate something funny going on—something well outside the state of chemical equilibrium—on a potentially hospitable planet. Perhaps our astronomy colleagues should be forgiven for their enthusiasm in declaring that humanity is on the brink of discovering alien life.

But haven't we heard this before? Didn't President Clinton announce in 1996 that a Martian meteorite recovered in Antarctica [1] “speaks of the possibility of life” on Mars? (No, it turned out to be mineralic artifacts.) Wasn't some “alien” arsenic-based life discovered recently in Mono Lake, California [2]? (No, it's a familiar proteobacterium struggling to survive in a toxic environment.) Didn't Craig Venter and his colleagues recently create a synthetic bacterial cell [3], “the first self-replicating species we've had on the planet whose parent is a computer”? (No, its parent is *Mycoplasma mycoides* and its genome was dutifully reconstructed through DNA synthesis and PCR amplification.)

Why are we so confused (or so lonely) that we have such trouble distinguishing life from non-life and distinguishing our biology from another? A key limitation is that we know of only one life form, causing us to regard life from that singular perspective (Figure 1). We see life as cellular, with a nucleic acid genome that is translated to a protein machinery. Life self-reproduces, transmits heritable information to its progeny, and undergoes Darwinian evolution based on natural selection. Life captures high-energy starting materials and converts them to lower-energy products to drive metabolic processes. Life exists on at least one temperate, rocky planet, where it has persisted for about four billion years. There are likely to be tens of thousands of “habitable” planets within a thousand light years of Earth, and more than a billion such planets in our galaxy, so *surely* (say the astronomers) we are not alone.

**Figure 1:**
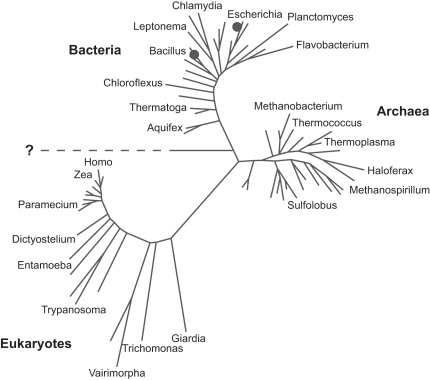
Phylogenetic tree of life based on small-subunit ribosomal RNA sequences, showing representative species from each of the three kingdoms (compiled by Pace [**11**]). The root of the tree is indicated by a horizontal line. The locations on the tree of *Halomonas* sp. (GFAJ-1) [2] and *Mycoplasma mycoides* (JCVI-syn1.0) [3] are indicated by black circles adjacent to *Escherichia* and *Bacillus*, respectively.

## Rolling the Dice

What, in fact, is the probability that a temperate, rocky planet will generate life? Science cannot say. That is because, based on the one known example of obscure origins, even a Bayesian would not want to assign a probability to such an event. The probability assessment would be more meaningful if there were even one more genuine example of life, whether discovered in space, on Earth, or in a test tube. If that entity had all of the properties of terrestrial life described above, then one would conclude that, indeed, we are not alone. But what if the entity had only some of those properties? What if it could self-reproduce, directing the assembly of progeny of identical composition, but could not evolve new functions? What if it consisted of complex chemical processes within a cellular compartment but had no basis for maintaining heritable genetic information? What if it had all of the properties of life but was descended from our own life form rather than derived from an independent origin?

When faced with such real or hypothetical situations regarding alternative life, it is useful to frame the question in terms of information: How many heritable bits are involved, and where did they come from? (Box 1) Biological systems are distinguishable from chemical systems because they contain components that have many potential alternative compositions but adopt a particular composition based on the history of the system. In this sense biological systems have a molecular memory (genotype), which is shaped by experience (selection) and maintained by self-reproduction. One can count the number of bits in this molecular memory, for example, up to two bits per base pair for a nucleic acid genome. The bits accrue as potential alternative compositions are excluded and specific compositions are adopted. More formally, the number of bits is calculated as log_2_ of the number of potential compositions divided by the number of realized compositions. One must count only those bits that accrue within the system, not those that were evolved elsewhere and bestowed upon the system for free.

**Box 1. Counting Bits**

When evaluating potential examples of alternative life, one should look at the genetic material and ask the following questions regarding its information content. The aim is to determine how many heritable bits are involved, excluding those that did not arise within the genetic system.

**Q1.** How many different types of subunits (x) does the genetic material contain? For a binary polymer, x = 2; for a nucleic acid polymer, x = 4 (A, T/U, G, C).

**Q2.** What is the length (n) of the genetic material? Count only those positions that can potentially be occupied by different types of subunits.

**Q3.** Do all of the possible compositions have the same prior probability of occurrence? Some combinations may be less probable if one of the subunits is less abundant or less frequently incorporated into the genetic material. Some combinations may be disfavored for chemical or biochemical reasons (e.g., long runs of G residues in DNA).

The number of possible compositions is x^n^. If these are all equally probable, then each has a prior probability of occurrence of x^−n^, and the information content (number of bits) associated with a particular realized composition is log_2_(x^n^). This can also be expressed as 2^#bits^ = x^n^. For a binary polymer, #bits = n; for a nucleic acid polymer, #bits = 2n.

If the various compositions do *not* have the same prior probability of occurrence, then the information content associated with a particular realized composition must be calculated based on its prior probability of occurrence (*p*_k_), which ranges from 0 to 1. The information content (number of bits) associated with a particular realized composition is −log_2_(*p*_k_).

**Q4.** Was a portion of the composition of the genetic material determined outside the genetic system? Some of the composition may have evolved in a different system and was merely available for the taking. Do not include preexisting information when counting bits because its prior probability of occurrence is 1, and therefore its information content with respect to the genetic system being considered is −log_2_(1) = 0.

Suppose one has a molecule that self-duplicates indefinitely, directing the ordered assembly of building blocks to produce additional copies of itself. That would be an interesting chemical process, but unless there is the opportunity for alternative compositions to arise and similarly reproduce—that is, for Darwinian evolution to occur—the bit content would be zero. Suppose one has a complex reaction cascade, perhaps even an autocatalytic cycle, contained within a growing and dividing physical compartment. That too would be an interesting chemical process, but it would not involve any heritable bits. Suppose one uses the genetic information from a preexisting biological organism to construct a facsimile that, going forward, can evolve alternative compositions. New bits could accrue within such a system, but all of the bits that were provided at the outset would have derived from the preexisting organism. To be considered a new life form, the majority of bits must be self-derived.

## Pathways to Life

**Figure 2:**
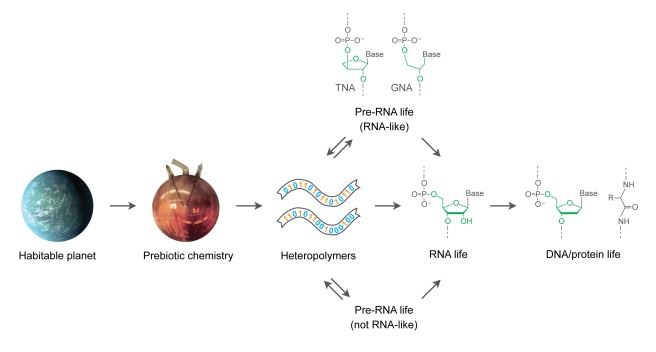
Potential pathways to life on Earth, beginning with a habitable planet and ending with DNA/protein-based life. The planet is an artist's conception of the recently discovered [12] “Earth-like” planet Kepler-22b (image courtesy of NASA/Ames/JPL-Caltech). Prebiotic chemistry is represented by the Miller-Urey spark-discharge apparatus (modification of photo by Ned Shaw, Indiana University). Two examples of hypothesized pre-RNA life are shown, based on either threose nucleic acid (TNA) or glycol nucleic acid (GNA).

In principle, there are two pathways by which a new life form can arise: either directly from chemistry or spun off from some other biology. If life arises from chemistry, as is thought to have occurred on the primitive Earth (Figure 2), then it begins with zero heritable bits and organizes into a bit-generating system. Following an era of prebiotic chemistry, perhaps reaching a high level of chemical complexity, molecular memory arises. Molecules of variable composition begin to replicate, mutate, and evolve in a Darwinian manner. If, instead, life arises from another life form, then it may have a privileged beginning, benefitting from a chemical environment that has been shaped by preexisting life. A new life form may grow on the spoils of prior life with no carryover of bits, or it may descend from prior life with some carryover of bits into a different genetic system. The latter type of transition is thought to have occurred during the early history of life on Earth when RNA-based life gave way to DNA/protein-based life [4]. That transition likely involved a substantial transfer of bits that had accrued within RNA and were ported over to DNA through what might be termed The Great Reverse Transcription. We see echoes of those ancestral bits in the sequences of ribosomal RNA, tRNA, and possibly other contemporary RNAs that are present across all three kingdoms of life.

It has been suggested that RNA-based life was preceded by yet another life form [5], perhaps based on a nucleic-acid-like genetic molecule such as threose nucleic acid (TNA) [6] or glycol nucleic acid (GNA) [7]. There does not appear to be any trace of pre-RNA life in contemporary biology, although maybe we do not know how to look. It is conceivable, for example, that some of the deeply conserved bits in contemporary RNAs are the double echo of information that was carried from pre-RNA life to RNA life and then to DNA/protein-based life. It also is possible that there was life on Earth prior to RNA but that it had no chemical resemblance to RNA. Information from such a pre-RNA genetic molecule could have been translated into RNA through a machinery analogous to the ribosome, but with RNA as the output rather than the input. Alternatively, pre-RNA life may have facilitated the emergence of RNA life, but with no transfer of information. Pre-RNA life may have generated RNA molecules as a metabolic product, and eventually the RNA molecules began replicating and evolving on their own.

## Getting Started

The above discussion emphasizes that there are many possible routes by which one life form can give rise to another, but it does not address the question of how an initial life form arises in the first place. A key question to ask is: What is the minimum number of bits it takes to provide a replicating, evolving system that has the ongoing capacity to accrue more bits? That will depend on the chemical nature of the first self-replicating molecules and the resources that are available in its environment. When astronomers speak of habitable zones they refer to a planetary orbit that is of appropriate distance from a star to maintain liquid water on the planetary surface. This may be too restrictive or too generous a definition, depending on your point of view [8]. Perhaps life can exist in a non-aqueous environment, although there is little data to support this conjecture. Conversely, even a temperate little pond of water, salts, and dilute organics may be insufficient for life to originate. The pond would need to accumulate heteropolymers of variable composition, including some that could replicate and provide the basis for molecular memory, and it is not clear whether this is a common or exceedingly rare occurrence.

In terms of bits, a binary heteropolymer that contains 10 subunits would have 1,024 potential compositions, and any particular composition would represent up to 10 bits of heritable information. It seems doubtful that there is any heteropolymer for which 10 bits is enough to get started. Now suppose that it is a quaternary heteropolymer (like RNA) that is assembled into chains of 40 subunits. Then there would be 10^24^ possible compositions, and any particular composition would represent up to 80 bits of heritable information. An RNA biochemist might argue (see below) that this is just enough to get started. However, to represent all of these combinations as even a single copy would require that the little pond contain 27 kilograms of RNA, which seems highly implausible. Finally, suppose there are millions of winning compositions of 40 subunits, any of which could self-replicate and initiate Darwinian evolution. Then even a lucky milligram of RNA might contain the seed of life.

In the laboratory one can prepare milligram quantities of random-sequence RNA molecules containing 40 or more subunits. One can provide an endless supply of activated nucleotide building blocks and control all aspects of the reaction conditions. Stacking the deck in this way, why haven't we witnessed the origin of RNA life experimentally? Because even a lucky milligram of RNA is insufficient. In order for a seed copy of replicating RNA to germinate, it must produce additional copies of itself faster than the existing copies become degraded, and it must operate with sufficient fidelity that the accurate copies are not overwhelmed by error copies [9]. The requisite rate and fidelity of replication might reasonably be achieved in a pure reaction system that contains only the replicator and its corresponding building blocks. However, in a complex mixture of almost entirely mismatched parts, what process singles out the rare matching components? Darwinian evolution can enrich one molecule in a milligram, but before the onset of Darwinian evolution there would be only chemistry, and the chemistry of complex mixtures of cross-interacting molecules is very messy.

One way to avoid complex mixtures would be for each replicating RNA, together with its corresponding building blocks, to be spatially isolated within a separate compartment. These compartments need not be “cells,” but at least separate locales to avoid crosstalk among the replicators and to allow the fruits of their labor to accumulate locally for their own benefit. The need for compartments places additional restrictions on the nature of the environment, although at a minimum a “compartment” could be as simple as a non-covalent assemblage of the replicator and its components. Again it comes down to bits. How many bits of specific chemical organization are required to exclude the molecular riff-raff and allow a fledgling replicator to begin evolving? If 80 bits are required and there are a million such 80-bit combinations, then there would need to be 10^18^ separate locales of suitable composition to provide a reasonable opportunity for life to begin. If each suitable locale is a bubble in the sea, then the origin of life may not be a rare event, but if each is a different habitable planet in our galaxy, then even among a billion such planets, we may be very much alone.

## Second Life?

**Figure 3:**
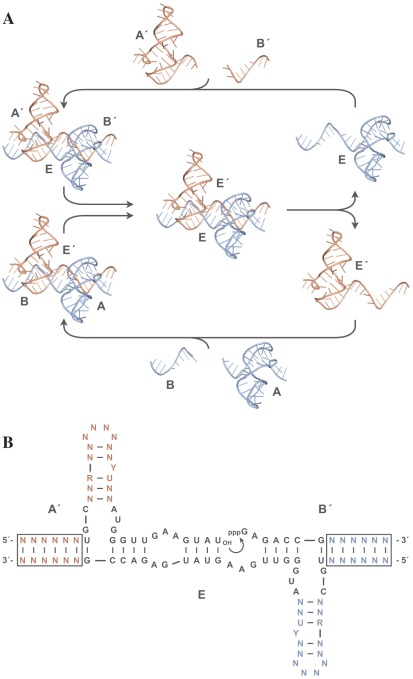
Self-replicating RNA enzymes that are capable of undergoing Darwinian evolution. (A) The replication cycle involves paired RNA enzymes (E in blue, E′ in orange) that catalyze each other's synthesis by joining two corresponding oligonucleotide substrates (A′ and B′ to form E′, A and B to form E). Each substrate contains six nucleotides of variable sequence that are recognized by Watson-Crick pairing to the enzyme. The tertiary structure is based on homology modeling to the crystal structure of the L1 ligase RNA enzyme [13]. (B) Sequence and secondary structure of the E·A′·B′ complex, with the two genetic regions (boxed) and two corresponding regions within the functional domain of the enzyme shown in color, and with the immutable nucleotides that are essential for replication shown in black. Curved arrow indicates the site of ligation of A′ and B′ to form E′. The E′·A·B complex has reciprocal composition.

My laboratory recently described an example, outside of biology, of a chemical system that can undergo Darwinian evolution in a self-sustained manner [10]. “Self-sustained” in this context means that all of the bits necessary for the system to undergo Darwinian evolution are part of the system that is evolving. The chemical system involves pairs of RNA enzymes that catalyze each other's synthesis by joining together two oligonucleotide building blocks (Figure 3A). Each of the two building blocks can adopt thousands of potential alternative compositions and thus can be joined to form millions of different combinations. Each of the many possible combinations can self-replicate and transmit compositional information to its progeny. Variants that replicate most efficiently grow to dominate the population until new, more advantageous variants arise to supplant their predecessors in a never-ending Darwinian battle for survival.

The population of evolving RNA enzymes constitutes a synthetic genetic system, but it is limited in two important respects. First, the molecules contain only 24 bits (12 base pairs) of heritable information to encode function. Second, replication depends on 60 bits (30 defined nucleotides) that are provided at the outset and are not subject to mutation and selection (Figure 3B). Thus of the 84 total bits required for the system to replicate and evolve, only about one-fourth can be counted as part of the system's molecular memory. The synthetic genetic system is not a new life form because it operates mostly on borrowed bits.

## Prospects

Someday the threshold may be crossed in which an alternative genetic system contains more heritable bits than the number of bits required to initiate its operation. Crossing that threshold is a reasonable criterion for what would constitute a new form of life. A life form that arises directly from bit-free chemistry would be considered “new” from the outset, while one that derives from a biological cell would have a long way to go before reaching the threshold. Between these two extremes lie the possibilities of starting with a modest number of bits, whether by the luck of combinatorial chemistry or derived from preexisting life, then accruing enough bits within the system to be regarded as new life. Perhaps the first true alternative to terrestrial biology will be found on an extrasolar planet, in a rock from Mars, or within an extreme environment on Earth. More likely, it will be the handiwork of an intelligent species that has discovered the principles of Darwinian evolution and learned to devise chemical systems that have the capacity to generate bits on their own.

## Abbreviations

GNA: glycol nucleic acid
TNA: threose nucleic acid
